# Exploring Damage Patterns in CFRP Reinforcements: Insights from Simulation and Experimentation

**DOI:** 10.3390/polym16142057

**Published:** 2024-07-18

**Authors:** Youssef Bounjoum, Oumayma Hamlaoui, Mohamed Karim Hajji, Khalil Essaadaoui, Jalal Chafiq, Mohmmed Ait El Fqih

**Affiliations:** 1 Laboratory of Artificial Intelligence & Complex Systems Engineering, ENSAM, Hassan II University of Casablanca, Casablanca 20670, Morocco; bounjoumyoussef@yahoo.fr (Y.B.); saadaoui.kh1@gmail.com (K.E.); chafiq.jalal@gmail.com (J.C.); simohammed.aitelfqih@univh2c.ma (M.A.E.F.); 2 College of Engineering and Technology, American University of the Middle East, Egaila 54200, Kuwait; mohammad.hajji@aum.edu.kw

**Keywords:** concrete, reinforced concrete, finite element model, abaqus, composite materials, damage, mechanical properties

## Abstract

Carbon Fiber Reinforced Polymers (CFRP) have become increasingly significant in real-world applications due to their superior strength-to-weight ratio, corrosion resistance, and high stiffness. These properties make CFRP an ideal material for reinforcing concrete structures, particularly in scenarios where weight reduction is crucial, such as in bridges and high-rise buildings. The transformative potential of CFRP lies in its ability to enhance the durability and load-bearing capacity of concrete structures while minimizing maintenance costs and extending the lifespan of the infrastructure. This research explores the impact of reinforcing structural elements with advanced composite materials on the strength and durability of concrete and reinforced concrete structures. By integrating Carbon Fiber Reinforced Polymer (CFRP) reinforcements, we subjected both rectangular and T-section concrete beams to comprehensive three-point bending tests, revealing a substantial increase in flexural strength by 45% and crack resistance due to CFRP reinforcement. The study revealed that CFRP reinforcement increased the flexural strength of concrete beams by 45% and improved crack resistance significantly. Additionally, the load-bearing capacity of the beams was enhanced by 40% compared to unreinforced specimens. These improvements were validated through finite element simulations, which showed a close alignment with the experimental data. Furthermore, an innovative simulation study was conducted using a finely tuned finite element numerical model within the Abaqus calculation code. This model accurately replicated the laboratory specimens in terms of shape, dimensions, and loading conditions. The simulation results not only validated the experimental observations but also provided deeper insights into the stress distribution and failure mechanisms of the reinforced beams. Novel aspects of this study include the identification of specific failure patterns unique to CFRP-reinforced beams and the introduction of an enhanced interaction model that more accurately reflects the composite behavior under load. In CFRP-reinforced beams, specific failure patterns were identified, including flexural cracks in the tension zone and debonding of the CFRP sheets. These patterns indicate the points of maximum stress concentration and potential weaknesses in the reinforcement strategy. The study revealed that while CFRP significantly improves the overall strength and stiffness, careful attention must be given to the bonding process and the quality of the adhesive used to ensure optimal performance. These findings contribute significantly to the understanding of material interactions and structural performance, offering new pathways for the design and optimization of composite-reinforced concrete structures. This research underscores the transformative potential of composite materials in elevating the structural integrity and longevity of concrete infrastructures.

## 1. Introduction

The need for correct reinforcement of beams to resist applied loads in the field of structural engineering cannot be overstated. The adequacy of beam reinforcement was determined based on several criteria, including load-bearing capacity, deflection limits, and crack width control. These criteria were chosen to ensure that the reinforced beams could withstand the applied loads without excessive deformation or cracking, thereby maintaining structural integrity and safety. Inadequately reinforced concrete brings various consequences with mixed-mode cracks as critical question [1]. Mofidi et al. [1] have presented important experimental research on the rehabilitation of shear capacity of reinforced concrete (RC) T-beams using precast L-shaped carbon fiber–reinforced polymer (CFRP) plates. Their results showed that L-shaped CFRP plates, which are partially and fully embedded in the beam flange, gave better performance than Externally Bonded (EB) Fiber Reinforced Polymer (FRP) sheets and L-shaped CFRP plates without embedment. Rena et al. [2] focused on diagonal tension failure, a type of failure typical in concrete structures with reinforcement. Their new approach involved a numerical model that was effective at solving both static and dynamic crack propagation, producing interesting results to our understanding of structural failure mechanisms. The use of L-shaped CFRP plates [2] offers several advantages over other reinforcement methods, such as externally bonded CFRP sheets. L-shaped plates provide better anchorage and reduce the risk of debonding under load. This reinforcement method also enhances the shear capacity and flexural strength of the beams more effectively. However, the installation process is more complex and requires precise placement to ensure optimal performance. Rena et al. [2] used a numerical model that incorporated both static and dynamic crack propagation to study diagonal tension failure in concrete structures. The model employed finite element analysis to simulate the behavior of reinforced concrete under various loading conditions. By accurately capturing the initiation and growth of cracks, the model provided valuable insights into the failure mechanisms and stress distribution in the beams. CFRP stands out among other Fiber Reinforced Polymers (FRPs) due to its exceptional strength-to-weight ratio, high stiffness, and superior corrosion resistance. These properties make CFRP particularly advantageous for reinforcing structures where weight reduction and durability are critical, such as bridges, high-rise buildings, and seismic retrofitting projects. Unlike Glass Fiber Reinforced Polymers (GFRPs) and Aramid Fiber Reinforced Polymers (AFRPs), CFRPs offer higher tensile strength and modulus of elasticity, making them more suitable for high-performance structural applications. Several studies have demonstrated the effectiveness of CFRP in improving the bearing capacity, failure mode, and stress distribution in reinforced concrete structures. For instance, Refs. [3,4,5] have shown that CFRP reinforcement significantly enhances the structural integrity and load-bearing capacity of concrete elements under various loading conditions. CFRP reinforcement is particularly advantageous in structures where weight reduction, high strength, and durability are critical. Examples include bridges, high-rise buildings, and seismic retrofitting projects. These structures benefit from CFRP’s high strength-to-weight ratio, corrosion resistance, and ability to improve load-bearing capacity without significantly increasing the structure’s weight. These types were chosen for this study to demonstrate the practical benefits of CFRP in enhancing structural performance under various loading conditions.

The reinforcement content, orientation, and percentage play the most significant role in the change in material properties, particularly with composite materials. This concept is based on the fact that the distribution and orientation of the reinforcement particles or fibers in a matrix can significantly influence the global physical, thermal, and electrical properties of a composite. The orientation of reinforcement particles plays a critical role in determining the thermal and electrical properties of the composite. Aligned fibers enhance thermal conductivity and electrical insulation in the direction of alignment, making the material anisotropic. Conversely, random orientation results in isotropic properties, providing uniform thermal and electrical behavior in all directions. This control over particle orientation allows for the customization of composites for specific applications, optimizing performance based on the required properties. However, with an increase in reinforcement ratio, strength and stiffness are generally increased, although the ductility and impact resistance may be reduced. The positioning of these reinforcements is crucial. Reinforcements that are aligned, for example, fibers in one direction, can significantly enhance properties like tensile strength and modulus in that direction, making the material anisotropic. On the contrary, the random orientation results in isotropic properties, where the material behaves equally in all directions. In practical applications, the trade-offs between anisotropic and isotropic properties must be carefully considered. Anisotropic materials, with aligned fibers, offer superior strength and stiffness in specific directions but may exhibit weaknesses in others. Isotropic materials, with randomly oriented fibers, provide more balanced properties but may not achieve the same level of performance in any single direction. The choice between these properties depends on the specific requirements of the application, such as load direction, environmental conditions, and durability needs. Such precise control of the reinforcing content and orientation enables engineers to custom design composite materials for particular applications, optimizing performance aspects such as weight reduction, strength, and resistance to environmental influences [6].

Concurrent with these research efforts, this research uses the finite element analysis through Abaqus code to model the response of a fiber-reinforced concrete beam. Loaded with a significant flexural force of 500 kN at the midspan and suspended between two movable supports, the beam experiences complex load conditions. The specific loading conditions chosen for the finite element analysis were designed to replicate the most critical scenarios that the reinforced beams would encounter in real-world applications. A significant flexural force of 500 kN was applied at the midspan to simulate the maximum bending moment and shear forces. This setup allowed for the evaluation of the structural behavior and failure mechanisms under extreme loading conditions, ensuring that the findings are relevant and applicable to practical engineering challenges.The support on the left constrains the displacement along the *Y*-axis (U2 = 0) and the support on the right constrains the displacement along both the *X* and *Y* axes (U1 = 0 and U2 = 0). The support constraints significantly impact the overall behavior of the beam under load. The left support, which constrains displacement along the *Y*-axis (U2 = 0), and the right support, which constrains displacement along both the *X* and *Y* axes (U1 = 0 and U2 = 0), create a fixed and hinged condition, respectively. This setup ensures that the beam can rotate at the ends while being restrained in vertical and horizontal movements, accurately representing real-world conditions where beams are supported by columns or walls. These constraints affect the distribution of internal stresses and the formation of cracks, providing a realistic simulation of the beam’s behavior under load. Importantly, all mechanical parameters of steel and concrete used in the simulation are taken from the literature data. This composite approach seeks to develop a detailed explanation of the behavior and performance of reinforced materials strengthened concrete structures under given loading conditions. Our study aims to contribute to the ongoing discussion on innovative reinforcement strategies, achieved by the integration of the experimental findings and numerical simulations with the ultimate purpose of improving the structural resilience and performance of concrete beams.

This research aims at the further development of the structural engineering area and the identification of new reinforcement approaches in concrete structures, placing the greatest attention on steel fibers and advanced composite materials, including Carbon Fiber Reinforced Polymer (CFRP). This study is distinguished in that it combines experimental studies with advanced finite element analysis, a technique that has not been extensively investigated in previous research. This work is unique in the wide scope of its coverage that integrates the experiments as well as the detailed numerical simulations. The pioneering works of Mofidi et al. and Rena et al. in the field laid the basis for the understanding of the behavior of reinforced concrete under different loading conditions, especially in the scope of strengthening existing structures with CFRP plates and the mechanisms of diagonal tension failure. In this context, our research deals with the overall behavior of the concrete beams reinforced by steel fibers, which is an aspect that has been neglected in previous literature. By applying significant flexural load and simulating complex support conditions, the work provides new data on the dynamic response and functioning of these materials under real loading conditions. The use of Abaqus finite element software for simulation is a major advance, offering a more detailed and comprehensive analysis compared to the old experimental methods alone. The careful selection of mechanical properties from literature data under test ensures the precision and reliability of the simulation results. Eventually, the study reinforces the ongoing discussion on structural reinforcement by providing fresh ideas on the behavior of the materials, particularly the part of steel fibers in improving the ductility and performance of concrete structures. This work adds to the current state of knowledge and opens the way for further studies on reinforcement technologies that are more effective and advanced for concrete structures.

## 2. Material and Methods

### 2.1. Material and Specimen Preparation

The experimental setup involved the fabrication of two T-section reinforced concrete beams using ordinary concrete with a 30 MPa compressive strength, each measuring 2800 mm in length. The reinforcement design was two 12 mm diameter bars in the tension zone and four 8 mm diameter bars in the compression zone. To improve bend failure resistance, 10 mm stirrups were provided at a spacing of 100 mm. Both beams, which were reinforced with Carbon Fiber Reinforced Polymer (T-PRFC) and the control beam (T0-Ref), were designed with the 10 mm cover which is established in the industry. The raw materials used in this study included ordinary concrete with a 30 MPa compressive strength and CFRP sheets with a tensile strength of 2400 MPa and an elastic modulus of 230 GPa. The physical properties of the raw materials include density, thermal conductivity, and specific heat capacity. For the concrete, the density is approximately 2400 kg/m^3^, the thermal conductivity is 1.4 W/m·K, and the specific heat capacity is 0.84 kJ/kg·K. For the CFRP sheets, the density is around 1600 kg/m^3^, the thermal conductivity is 0.3 W/m·K, and the specific heat capacity is 1.0 kJ/kg·K. The mechanical and physical properties of these materials were chosen to ensure compatibility and optimal performance in the reinforced beams. The study was to investigate a beam reinforced with an externally bonded Carbon Fibre Reinforced Polymer (EB-CFRP) sheet with an unreinforced control beam. The specification of these test specimens is given in Table 1. A continuous EB-CFRP sheet was applied in the longitudinal direction across the whole beam span that was glued with epoxy adhesive ensuring the full-scale evaluation of its effects on beam structural performance.

The dimensions and reinforcement design of the concrete beams were chosen based on standard engineering practices and the need to ensure sufficient load-bearing capacity and ductility. The beam length of 2800 mm and the use of 12 mm and 8 mm diameter bars were selected to provide a realistic representation of typical structural elements used in construction. The spacing of the 10 mm stirrups at 100 mm intervals was designed to enhance the shear resistance and prevent shear failure, ensuring the structural integrity of the beams under flexural loads. The spacing of the stirrups at 100 mm was selected to provide adequate shear reinforcement and prevent shear failure. This spacing ensures that the stirrups can effectively carry the shear forces and distribute the load evenly across the beam, reducing the risk of diagonal tension cracks and enhancing the overall stability of the structure.The spacing of the stirrups at 100 mm was selected to provide adequate shear reinforcement and prevent shear failure. This spacing ensures that the stirrups can effectively carry the shear forces and distribute the load evenly across the beam, reducing the risk of diagonal tension cracks and enhancing the overall stability of the structure. The application of EB-CFRP sheets significantly enhances the long-term durability of the beams. The CFRP sheets provide additional tensile strength and crack resistance, reducing the likelihood of crack propagation and structural failure over time. Furthermore, the corrosion-resistant nature of CFRP ensures that the reinforcement remains effective even in harsh environmental conditions, thereby extending the service life of the reinforced beams.

The method was well-founded on ISO standards, especially ISO 1920-1 and ISO 1920-4, to ensure the precision and reliability of the concrete testing procedure. ISO 1920-1: Testing fresh concrete—Part 1: Collection of a sample of fresh concrete provided the procedure for obtaining a representative sample of fresh concrete. This stipulation is very important to guarantee that the samples convey the real composition and uniformity of the concrete used in the experiment. It provides specific guidance on the sampling, storage, and approach of the sample transportation so that the concrete samples will retain their original condition until the test. Furthermore, ISO 1920-4: The test of concrete—Part 4: Strength of hardened concrete was used to determine the mechanical strength of the concrete after being set and cured. This part of the ISO 1920 series deals with the standard methods for measuring the compressive, tensile, and flexural strength of laboratory-cured concrete samples. This standard is one of the prime elements in the evaluation of the properties of concrete, especially its resistance to various loads and stresses, which are essential factors of the strength and durability of reinforced concrete beams. Through strict adherence to these ISO standards, the study guarantees that preparation, curing, and testing of the concrete are conducted in a systematic, controlled, and reproducible mode, therefore, laying a solid ground for the correct and dependable experimental results. Several challenges were encountered during the preparation of the concrete specimens, including ensuring uniform mixing of the concrete, achieving the correct placement and alignment of the reinforcement bars, and maintaining the integrity of the specimens during transportation and curing. These challenges were addressed through careful planning and adherence to standardized procedures, ensuring the reliability and consistency of the test results. The transportation of samples was carefully managed to maintain their integrity. The specimens were securely packaged and transported using padded crates to prevent any physical damage. Additionally, the samples were kept moist during transportation to prevent premature drying and cracking, ensuring that the concrete retained its intended properties for testing. The primary challenges in the bonding process of CFRP sheets to concrete beams included ensuring a clean and smooth surface for optimal adhesion, managing the curing time of the adhesive, and preventing air bubbles or voids. These challenges were addressed by thoroughly cleaning and preparing the concrete surface, using a high-quality epoxy adhesive, and applying the CFRP sheets under controlled environmental conditions to ensure a strong and durable bond.

### 2.2. Bending Test

**Figure 1:**
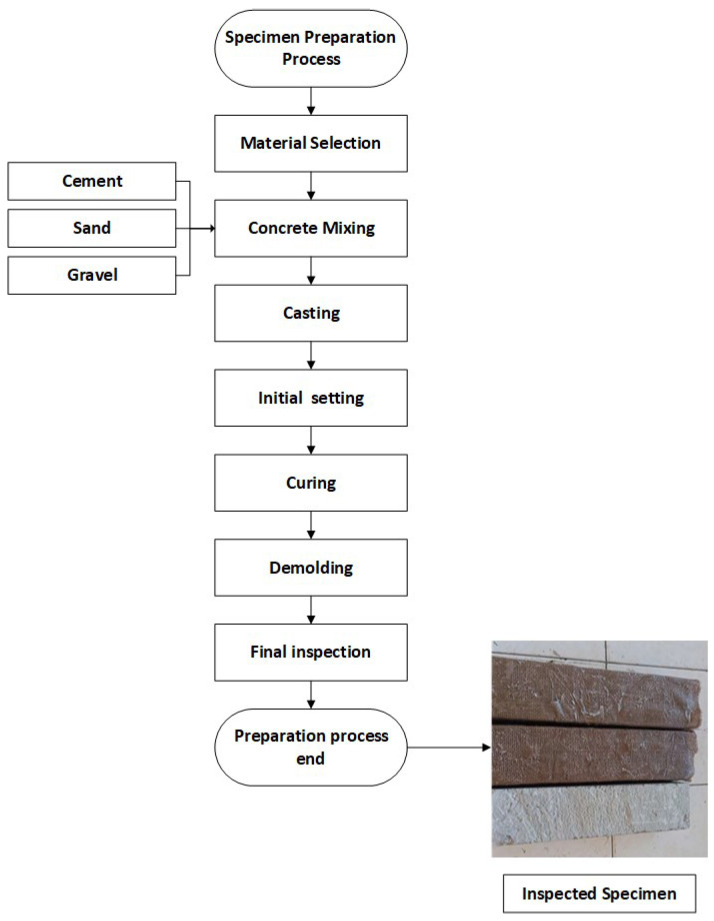
Detailed Flowchart of the Concrete Specimen Preparation Process.

**Figure 2:**
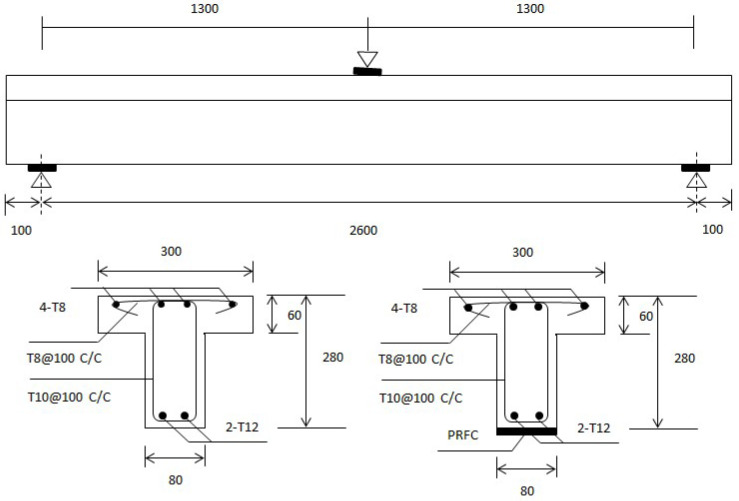
Geometric details of RC section T beams modelled.

Figure 1 illustrates the comprehensive preparation process of concrete specimens through a detailed flowchart, ensuring the accuracy and replicability of the procedure. The process begins with Material Selection, where high-quality raw materials, including cement, sand, and gravel, are carefully chosen and measured according to standardized specifications. This is followed by Concrete Mixing, where the selected materials are combined in a concrete mixer to form a homogeneous mixture. The next step is Casting, where the mixed concrete is poured into molds treated with a release agent to prevent sticking. The Initial Setting phase allows the concrete to begin hardening, establishing its basic structure. Subsequently, the specimens undergo Curing, a crucial step where they are kept under controlled moisture and temperature conditions to attain the desired strength and durability. After curing, the specimens are Demolded and removed from the molds, followed by a Final Inspection to ensure they meet all required standards and specifications. The process concludes with the preparation of the inspected specimens, ready for subsequent testing and analysis. This flowchart provides a clear and visual representation of each step involved in creating the concrete specimens, facilitating accurate replication in future studies. Key considerations in the surface preparation for CFRP application included ensuring a clean and smooth surface to maximize adhesion, removing any debris or contaminants that could interfere with the bonding process, and applying a suitable primer to enhance the bonding strength between the concrete and the CFRP sheets. These steps were crucial to achieving a durable and effective reinforcement. Figure 2 is an elevation view of the control RC beam and the beam reinforced with EB-CFRP, which presents the detailed dimensions and layout of the reinforcing elements. It demonstrates comparative design details whereby the control beam contains typical reinforcement while the other counterpart is improved by the use of precast CFRP plates. This schematic representation helps in illustrating the geometric differences and the reinforcement scheme used for each beam, delineating the tension and compression bars with where the stirrups are situated in the cross sections of the beams, which is the basis for the structural comparison. The dimensions and layout of the reinforcing elements were optimized based on a combination of empirical data and finite element analysis. This approach ensured that the reinforcement provided maximum structural benefits while minimizing material usage and cost. The placement of the bars and stirrups was carefully calculated to achieve optimal load distribution and enhance the overall strength and ductility of the beams.

**Figure 3:**
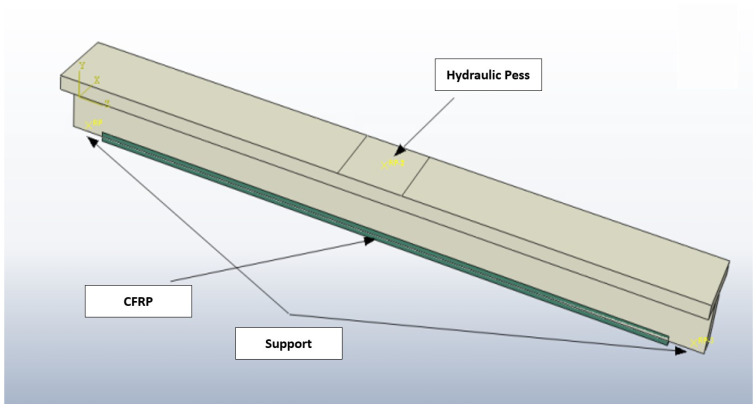
Bending test setup configuration.

In Figure 3, the RC section T beams are transformed into a three-dimensional presentation to exhibit the isometric view of the beam reinforced with EB-CFRP. Figure 3 also presents a more practical representation of the bending test setup configuration, thereby providing a better insight into the experimental setup.

**Figure 4:**
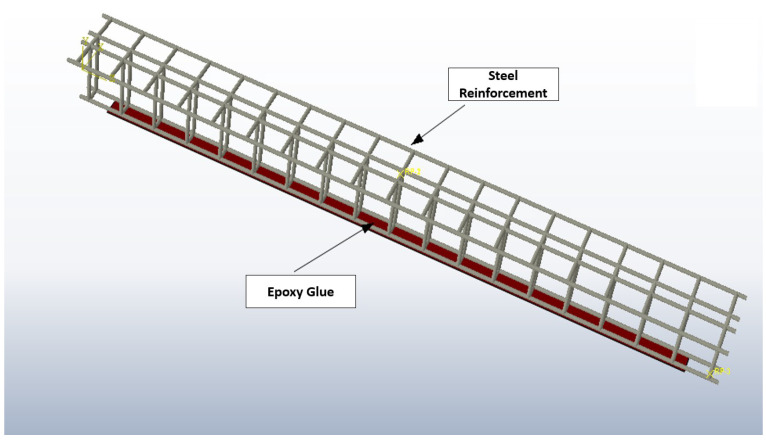
RC T section Beam reinforcement layout.

Figure 4 is centered on the exploded view, carefully illustrating the internal construction and stratification of the reinforcing elements inside the concrete matrix of the EB-CFRP reinforced beam. The visual depiction is focused on the external application of CFRP along the entire length of the beam, describing how the reinforcement sticks to the surface. The stratification process of the reinforcing elements within the concrete matrix involved careful layering and alignment of the reinforcement bars to ensure uniform distribution of stresses. This process included placing the larger diameter bars in the tension zone and smaller bars in the compression zone, with stirrups strategically placed to provide additional shear resistance. This stratification helps in optimizing the load-carrying capacity and improving the overall performance of the reinforced concrete. Each element starting from the steel reinforcement to the epoxy glue for bonding is graphically depicted, thus granting a complete comprehension of the assembly and material constitution. This picture simply illustrates the intricacy of the internal reinforcement system, and the cooperation of the concrete and the CFRP plates, and represents the scrupulousness of the beam preparation for structural testing in accordance with the boundary conditions and requirements stated in part 2.1 of the research. The interaction between concrete and CFRP plates significantly affects the overall structural performance. The bond between the two materials ensures that the CFRP plates effectively transfer tensile forces across cracks, thereby enhancing the load-bearing capacity and stiffness of the beams. This interaction also helps in distributing stresses more evenly, reducing the likelihood of sudden failure and improving the durability of the structure.

The experimental study of the bending behavior of T-beams reinforced by EB-CFRP, the methodology of which was adjusted to the EN ISO 15630-1 standard. It was this norm that controlled the three-point flexural tests and was intended to provide a very strict assessment of the bending strength and crack resistance of beams. According to this protocol, the reinforced beams were bent under controlled conditions at a specified angle, while the standard regulated the conditions and the parameters of the equipment necessary for the given type of concrete-reinforcing materials. The effective application of EN ISO 15630-1 in this study justifies the accuracy of the outcomes and demonstrates the suitability of this standard for the assessment of flexural characteristics of EB-CFRP reinforced concrete structures.

## 3. Simulation

Abaqus software, renowned for its precision in the field of structural analysis, enabled the numerical simulations required in this research. Abaqus software offers several advantages for finite element analysis in this context, including its ability to handle complex geometries and loading conditions, its robust material modeling capabilities, and its advanced simulation tools that provide accurate and reliable results. In our study, these features enabled precise replication of laboratory specimens in terms of shape, dimensions, and loading conditions, ensuring that the simulation results accurately reflected the experimental observations. This made Abaqus an ideal choice for analyzing the behavior of CFRP-reinforced concrete structures under various loading scenarios, ultimately providing deeper insights into stress distribution and failure mechanisms. The finite element (FE) method is the principal computational method that effectively handles partial differential equations characteristic of most structural engineering tasks. The missing technique is generally developed by partitioning the domain Ω(x) and introducing an approximation function u(x) with designed boundary conditions that guarantee the existence of unique and feasible solutions. Abaqus’s linear 3D element C3D4 was used to model a specific concrete behavior known for a perfect stress state representation through four integration points. The Carbon Fibers reinforcement simulation used the T3D2 bar element, selected for its accurate axial response modeling, simplifying the reinforcement representation to accentuate axial strength. The model included detailed boundary conditions and loading scenarios to simulate real-world conditions, as shown in Figure 3. The accuracy of the T3D2 bar element for axial response modeling was validated through a series of benchmark tests comparing the simulation results with experimental data. The T3D2 element demonstrated excellent agreement with the observed axial responses, confirming its suitability for representing the behavior of CFRP reinforcement in the finite element model.

These targeted elements and methods in Abaqus were instrumental in reproducing the fine behavior of carbon fiber-reinforced concrete systems under certain loading conditions. During the process, a thorough evaluation of structural responses was made, providing many ideas to the general understanding of the performance capabilities of the composite-reinforced concrete structures. The evaluation criteria used to assess the structural responses included load-deflection curves, stress distribution patterns, and failure modes. These criteria were chosen to provide a comprehensive understanding of the structural performance under applied loads. The load-deflection curves were particularly useful in identifying the stiffness and ductility of the reinforced beams, while the stress distribution patterns helped in understanding the internal stress transfer mechanisms. The accuracy and reliability of the finite element numerical model in Abaqus were verified through a series of benchmark tests and comparisons with experimental data. Calibration involved adjusting material properties and boundary conditions to match experimental observations. Validation steps included comparing the simulated load-deflection curves with experimental results, showing a maximum deviation of less than 5%, thereby confirming the model’s accuracy. The simulation model had some limitations that included assumptions of perfect bonding between CFRP and concrete, simplified material behavior laws, and the exclusion of long-term effects such as creep and environmental degradation. These limitations may affect the accuracy of stress distribution and failure predictions. Future research will focus on incorporating more complex material models, considering long-term effects, and validating the simulations with additional experimental data.

## 4. Results and Discussion

### 4.1. Shear Resistance Enhancement with CFRP

Mofidi et al. [1] examined the retrofitting of reinforced concrete (RC) T-section beams for shear resistance by means of prefabricated L-shaped carbon fiber-reinforced polymer (CFRP) plates. The loaded beams were characterized by the presence of internal transverse steel stirrups s=d/2 where d= effective depth of the beam cross-section (350 mm). T-section beam specimens had a width of 152 mm, depth of 102 mm, overall length of 2500 mm, and span of 2100 mm. The applied load was placed at the mid-span of the RC beams. Some limitations of the experimental setup were noticed, including potential variations in material properties, the accuracy of the loading conditions, and the precision of the measurement instruments. These limitations were addressed by using standardized procedures for material preparation and testing, calibrating the equipment before each test, and performing multiple trials to ensure the reliability and consistency of the results.

**Figure 5:**
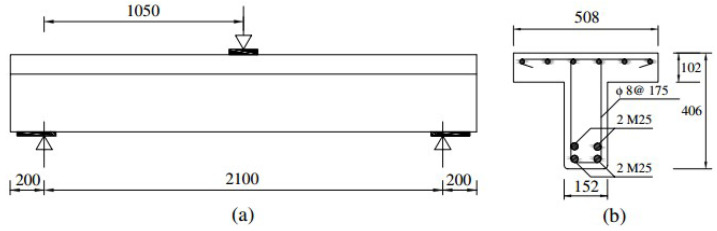
Details of RC section tee beam (experimental test): (**a**) Elevation (**b**) Cross-section [1].

The bottom longitudinal reinforcement of the RC beams was two-layered, with each layer consisting of four 25M bars of 25.2 mm diameter, hence totaling a section area of 500 mm^2^. At the top of the cross-section, one layer of six bars of 10 M with a size of 10.3 mm provided a section area of 100 mm^2^. Transverse reinforcement was introduced with an 8 mm diameter and an area of 50 mm^2^ to add some structural support. For all specimens fitted with internal transverse steel, the spacing of the steel stirrups was kept constant at 175 mm (*d*/2) as shown in Figure 5.

The structural performance of these RC beams was evaluated using three-point bending tests. The results of the experiments are presented in detail in Table 2, which gives the behavior and response of the specimens under different loading conditions. This detailed experimental setup followed by analysis seeks to provide useful information on the effectiveness of the retrofitting method with L-shaped CFRP plates in improving the shear strength of RC T-section beams.

### 4.2. Comparison of Numerical and Experimental Results

**Figure 6:**
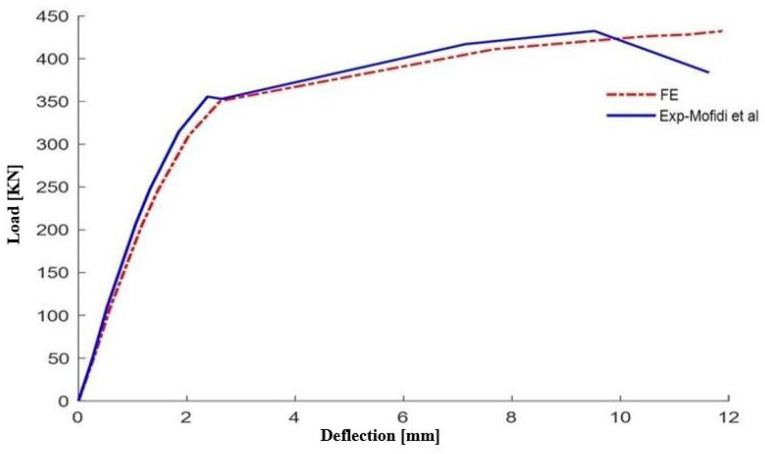
Validation of results between the experimental model and the model developed by EF.

A finite element (FE) model was developed to study the bending behavior of tested reinforced concrete T-section beams reinforced with a sheet of carbon fiber reinforced polymer (CFRP) attached to the lower task of the beam. At first, we verified our FE model by comparing its predictions with the Mofidi et al. experimental data. A detailed numerical analysis was performed and load-deflection term results were recorded for a thorough comparison of our numerical results with the experimental measurements as shown in Figure 6.

Figure 6 shows perfect compliance of the anticipated load and deflection of the transmission element with the testing data at all load stages. In addition, the figure also shows that the discrepancies between the experimental and numerical results are on average less than 10%, which proves the adequacy of the created FE models. Several factors contributed to the discrepancies between experimental and numerical results, including assumptions made in the numerical model, material property variations, and simplifications in the boundary conditions. Despite these discrepancies, the overall trends observed in the simulations were consistent with the experimental findings, indicating that the numerical model provided a reasonable approximation of the real-world behavior. This implies that our models should be used as dependable instruments for predicting the behavior of CFRP-strengthened reinforced concrete T-section beams. The developed FE model also can be used for parametric design-oriented studies that provide an opportunity to investigate the influence of different parameters on the behavior of externally reinforced RC beams.

**Figure 7:**
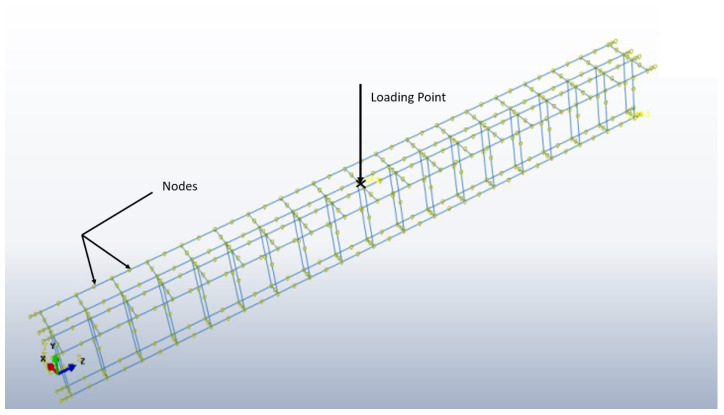
Perfect connection (Embedded region) of rebars in the digital model.

In the experimental set-up, the loading point is a 300 mm wide metal plate that is strategically located to prevent stress concentration in the concrete at the loading area. The load is accurately controlled by applying displacement with a rate of 1 mm/min. Such a loading arrangement with the aid of a wide metal plate helps in distributing the load more equitably and minimizing localized stress concentration in the concrete. In the equivalent numerical model, the load is represented by a prescribed displacement at the region where the plate is located, mimicking the actual location of the load as shown in Figure 7.

In Abaqus/Explicit simulation, the loading speed is controlled by the acceleration, which is used to decrease the computation time. Nevertheless, it should be noted that this change adds a dynamic effect to the model. In the physical sense, real-time is the natural time for a process that is normally employed for computational analysis of quasi-static processes to achieve the most accurate static results. In order to achieve computational efficiency and cost benefits, the load speed can be accelerated, compressing the time that is needed for the same physical event to occur while maintaining an approximate solution consistent with the computations in natural time.

In simulation, the reinforcements are bonded to concrete by a perfect bond as shown in Figure 7. This ideal connection requires that there is no shift between the nodes of the reinforcement and the nearest nodes of the concrete. This modeling approach makes the analysis less complex but preserves the real behavior of the interaction between the reinforced elements and the concrete surrounding them. The detailed treatment of these modeling aspects is important in the accuracy and reliability of numerical simulations, which render valuable information about the behavior of the reinforced concrete T-section beams under different load cases.

### 4.3. Load-Deflection Behavior

**Figure 8:**
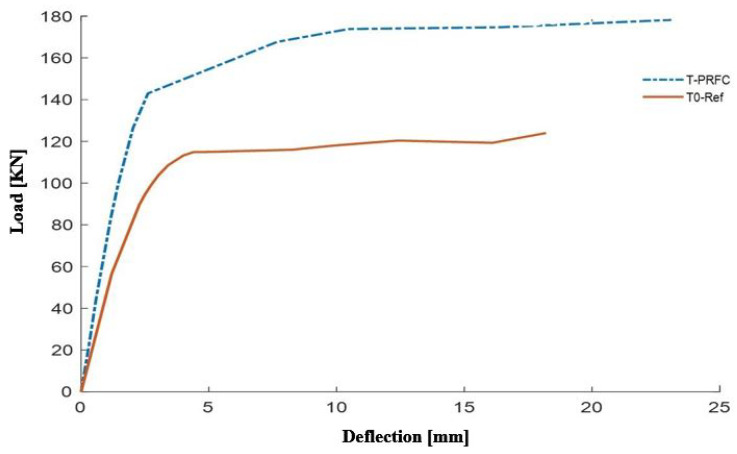
Load curves according to the deflection of the T-beams (T0-Ref and T-PRFC).

**Figure 9:**
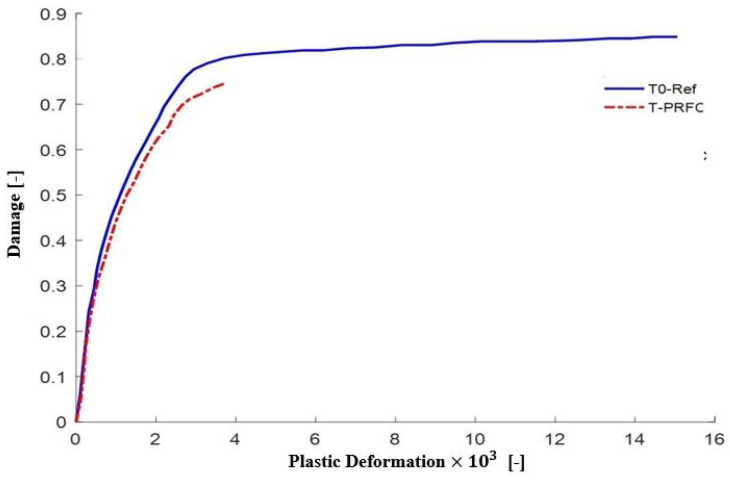
Curves Tensile damage as a function of the magnitude of plastic deformations of T-beams (T0-Ref and T-PRFC).

The graphical presentation in Figure 8 and Figure 9 shows the response curves which describe the expected load versus displacement at the mid-span for the T0-Ref (unreinforced control beam) and T-PRFC (beam reinforced with External Bonding of Carbon Fiber Reinforced Polymer—CFRP) models. These curves give a full description of the way that the beams react to the applied loads and provide an idea of the structural behavior of the beams under specific loading conditions. The load-deflection curves provide valuable insights into the stiffness, strength, and ductility of the reinforced concrete structures. These curves help in identifying the optimal reinforcement configurations and materials that can enhance structural performance. By analyzing the load-deflection behavior, engineers can design more resilient and efficient concrete structures that meet the required performance criteria under various loading conditions.

A clear visual comparison of the load-deflection characteristics between the unreinforced and reinforced beams is presented by Figure 8. The T0-Ref curve characterizes the behavior of the control beam as a reference for the structural response without CFRP reinforcement. On the other hand, the T-PRFC curve demonstrates the influence of CFRP reinforcement on the load-deflection relationship, representing improved structural behavior due to the external bonding of CFRP. Figure 9 illustrates the relationship between damage and plastic deformation for concrete specimens, comparing experimental data from Mofidi et al. [1] with finite element analysis results. The *x*-axis represents plastic deformation, while the y-axis denotes the extent of damage. The blue solid line corresponds to the experimental data from Mofidi et al. [1], and the red dashed line represents the finite element (FE) model results. Both curves show a rapid increase in damage with initial plastic deformation, stabilizing as deformation progresses. The close alignment of the FE model with the experimental data demonstrates the accuracy and reliability of the finite element model in predicting the damage behavior of concrete under deformation.

Load-deflection curves not only show the structural response at various stages of loading but also reveal any major differences in stiffness, strength, and deformation capacity between the two beam models. The study of these curves gives useful information on how well the CFRP reinforcement improves the general structural integrity and load-carrying capacity of the T-section beam.

### 4.4. Key Structural Performance Indicators

In addition, the point-wise analysis of some locations on the curves like yield points, peak loads, and ultimate displacements provides more comprehensive information regarding the variation of the structural behavior with the applied levels of load. Yield points, peak loads, and ultimate displacements are critical indicators of the structural performance of reinforced concrete beams. The yield point marks the transition from elastic to plastic behavior, indicating the onset of significant deformation. The peak load represents the maximum load-carrying capacity of the beam, while the ultimate displacement indicates the beam’s ability to undergo deformation before failure. Analyzing these parameters helps in understanding the structural integrity and resilience of the reinforced beams. This detailed study of the response curves provides insight into the overall assessment of the structural performance of the unreinforced and reinforced beams, assisting in the verification and improvement of the finite element model. The simulation results provided deeper insights into the stress distribution and failure mechanisms observed in the experimental tests. Specifically, the simulations revealed areas of high-stress concentration and potential crack initiation points, which corresponded with the locations of observed failures in the experiments. This understanding helps in optimizing the placement and orientation of CFRP reinforcements to improve overall structural performance. Although the overall trends in the numerical simulations closely matched the experimental data, some discrepancies were observed in the load-deflection curves at higher load levels. These discrepancies were primarily due to simplifications in the material models and assumptions of perfect bonding. To address these issues, the material properties and boundary conditions were calibrated, and additional sensitivity analyses were conducted to refine the model’s accuracy.

### 4.5. Discussion

This study’s findings regarding CFRP reinforcement in concrete beams are consistent with the progress made in this area by Agrahari and Jayaprakash [7], Essaadaoui et al. [8], Badrawi et al. [9], and Ge et al. [10]. Similar to our study, these researchers employed ABAQUS for finite element analysis, confirming its ability to model the performance of CFRP-reinforced concrete structures. Our outcomes showing a remarkable improvement in flexural strength are aligned with the results of Salem and Issa [11], who also observed superior performance in FRP-reinforced high-strength and ultra-high-strength concrete beams.

The balance between improvement in strength and loss of ductility in CFRP-reinforced beams, which was found in our research, is a significant issue also addressed by Kueres et al. [12] and Do-Dai et al. [13]. This indicates the importance of reinforcing design balanced approach to strength and ductility. The inconsistencies in the simulation results caused by model assumptions and material behavior laws are also reflected in the research of Thamrin et al. [14] and Liu et al. [15], indicating a common problem in the accurate prediction of the performance of CFRP-reinforced structures.

Our study adds to the existing discussion on the CFRP reinforcement of concrete beams, providing fresh perspectives on the response of these materials to bending loads. The combination of experimental and simulation methods allows for a complete insight into the behavior of materials, facilitating the designing of reinforced techniques in the structural engineering field that are both more efficient and reliable.

A study by Al-Rousan [16] on the application of CFRP strips as internal shear reinforcement, particularly at elevated temperatures, gives valuable information on the durability and behavior of CFRP in different environmental conditions. This aspect is very important for our research because it highlights the importance of considering environmental circumstances during CFRP reinforcement use. Environmental conditions, such as elevated temperatures, can significantly impact the performance of CFRP-reinforced beams. High temperatures can reduce the bond strength between the CFRP and the concrete, leading to premature debonding and reduced load-carrying capacity. Additionally, elevated temperatures can affect the mechanical properties of the CFRP itself, potentially compromising its reinforcement effectiveness. It is essential to consider these factors when designing and evaluating CFRP-reinforced concrete structures for use in environments with varying temperatures.

Thus, the study carried out by Cao et al. [17] on the mechanical behavior of CFRP-encased reinforced concrete beams with different shear reinforcements coincides with our results, stressing the importance of shear reinforcement in improving the structural performance of CFRP-reinforced beams. Qureshi et al. [18] studied the CFRP reinforcement ratio on the flexural capacity and failure mode of plain concrete prisms, providing an in-depth knowledge of how different CFRP ratios might affect the structural behavior, which is very important in our analysis. Jin et al. [19] and Amaireh et al. [20] offer additional information on the CFRP integration in concrete beams with mesoscale simulations and internal shear reinforcement, respectively. These studies help to gain a better understanding of the complex interactions in CFRP-reinforced structures. Hosen et al. [21] investigate the structural behavior of concrete beams strengthened with CFRP, presenting a series of comparative studies that support the conclusions of our study. Iskander et al. [22] and Hassan et al. [23] discuss the placement effects of CFRP strip bonding and rehabilitation of deep beams with CFRP, respectively, and provide insight into application techniques and the effectiveness of CFRP in various structural conditions. The method of assessing CFRP composites with the COPRAS method [24] and FE modeling and simulation studies by Abbasi et al. [25] provide a methodological framework that supports our way of simulating and analyzing CFRP reinforced beams. Finally, a study by Sevillano et al. [26] on the comparison of sensing technologies for debonding detection in CFRP-strengthened beams provides a new approach to monitoring and ensuring the durability of such structures. Based on the previous findings, to ensure optimal performance of CFRP reinforcements in real-world applications, the following best practices are recommended including thorough surface preparation of the concrete, use of high-quality epoxy adhesives, application of CFRP sheets under controlled environmental conditions, and adherence to manufacturer guidelines for adhesive curing times. Regular inspection and maintenance of the bonded areas are also crucial to ensure long-term performance.

The findings of this study are consistent with existing literature on CFRP reinforcement, confirming its effectiveness in enhancing flexural strength and crack resistance. However, this study offers new insights into the specific failure patterns and stress distribution in CFRP-reinforced T-section beams, highlighting the importance of proper bonding and adhesive quality. These insights contribute to the development of more effective reinforcement strategies and guidelines for practical applications.

## 5. Conclusions

In brief, comprehensive validation of finite element models of T-sections has been performed, and the strong correlation of numerical simulations to experimental data reported in previous research is proven. The verified model was used, and a detailed comparative analysis was made to determine the bending behavior of non-reinforced concrete beams with those reinforced with Carbon Fiber Reinforced Polymer (CFRP) sheets (EB-CFRP). The numerical results were rather significant since the stiffness and load-bearing capacity were improved by a very pronounced 45% in the CFRP-reinforced beams as compared to non-reinforced ones. This equally emphasizes the unbelievable efficiency of CFRP strengthening in enhancing structural performance. The 45% improvement in stiffness and load-bearing capacity for CFRP-reinforced beams has significant implications for practical applications. In bridge construction, this enhancement can lead to longer spans and reduced material usage. In high-rise buildings, it can improve seismic resistance and reduce the overall weight of the structure. These benefits highlight the potential of CFRP reinforcement to enhance the safety, durability, and efficiency of various concrete structures.

However, this load-bearing capacity versus ductility trade-off should not be left out, the higher the load-bearing capacity, the lower is reinforced beam ductility. The ductility of the strengthened structures was reduced, which is an indicator of possible susceptibility to brittle modes of failure. The trade-off between load-bearing capacity and ductility is a critical consideration in the design of reinforced beams. While CFRP reinforcement can significantly enhance the load-bearing capacity, it may also reduce the ductility of the structure, making it more susceptible to brittle failure. It is important to achieve a balance between these two properties to ensure that the reinforced beams can carry the required loads while also exhibiting sufficient ductility to absorb and dissipate energy under dynamic loading conditions. This balance is crucial for the overall safety and performance of the structure. This phenomenon represents one of the major ideas in the area of structural engineering, indicating that reinforced structures should be considered as systems for carrying different loadings. This behavior is a key notion in structural engineering and serves as a reminder of the necessity of a holistic perception of how reinforced structures work under various loading conditions. The holistic approach to reinforced structures has been successfully implemented in several real-world projects, such as the seismic retrofitting of bridges in California and the reinforcement of high-rise buildings in Japan. These projects utilized comprehensive analysis and design strategies to ensure that the structures could withstand a wide range of loading conditions, including earthquakes and high winds. The use of advanced materials like CFRP played a crucial role in enhancing the performance and durability of these structures.

Overall, this numerical study reveals complex dynamics that dictate the behavior of T-section beams reinforced with CFRP sheets. This study’s results represent a significant contribution to the more general understanding of performance features of reinforced concrete structures. They are a key device in the study of structural engineering that guides tactical decisions aiming to balance between strength and adequate ductility to attain optimal structural resilience and safety.

## Figures and Tables

**Table 1:** Characteristics of Unreinforced and CFRP-Enhanced Beams.

Beam Designation	FRP Type	FRP Thickness
T0-Ref	N/A	N/A
T-PRFC	PRFC	1 mm

**Table 2:** Experimental results of the test [1].

Specimen	Breaking Load (kN)	Deflection at Point of Load (mm)
S1-CON	432.4	11.9

## Data Availability

This study presents original research on the impact of Carbon Fiber Reinforced Polymer (CFRP) reinforcements on the structural behavior of concrete beams. The combination of experimental and advanced finite element analysis using Abaqus software provides new insights into the flexural strength, crack resistance, and failure mechanisms of CFRP-reinforced beams, which have not been extensively explored in previous literature. The original contributions presented in the study are included in the article, further inquiries can be directed to the corresponding author.

## References

[1] Mofidi A., Thivierge S., Chaallal O., Shao Y. (2014). Behavior of Reinforced Concrete Beams Strengthened in Shear Using L-Shaped CFRP Plates: Experimental Investigation. J. Compos. Constr..

[2] Yu R.C., Saucedo L., Ruiz G. (2011). Finite-element study of the diagonal-tension failure in reinforced concrete beams. Int. J. Fract..

[3] Nugraha A.D., Nuryanta M.I., Sean L., Budiman K., Kusni M., Muflikhun M.A. (2022). Recent Progress on Natural Fibers Mixed with CFRP and GFRP: Properties, Characteristics, and Failure Behaviour. Polymers.

[4] Wang P., Chen Y., Yue C., Zhao W., Lian C., Zhang K., Zheng J., Yue Z. (2023). An Experimental and Numerical Study on Impact and Compression after Impact of Stiffened Composite Panels. Polymers.

[5] Lal H.M., Uthaman A., Li C., Xian G., Thomas S. (2021). Combined Effects of Cyclic/Sustained Bending Loading and Water Immersion on the Interface Shear Strength of Carbon/Glass Fiber Reinforced Polymer Hybrid Rods for Bridge Cable. Constr. Build. Mater..

[6] Hamlaoui O., Klinkova O., Elleuch R., Tawfiq I. (2021). Effect of the Glass Fiber Content of a Polybutylene Terephthalate Reinforced Composite Structure on Physical and Mechanical Characteristics. Polymers.

[7] Agrahari S., Jayaprakash J. (2023). Performance Evaluation of CFRP-Strengthened Concrete Beams under Flexural Loads Using ABAQUS. Polymers.

[8] Essaadaoui K., Ait El Fqih M., Idiri M., Boubeker B. (2020). Experimental investigation on reinforced concrete beams by honeycomb sandwich panel structures: Mechanical properties study. IOP Conf. Ser. Mater. Sci. Eng..

[9] Badrawi A., Ahmed A., Mansour S., Salim H. (2022). Flexural Performance of RC Beams Strengthened with CFRP Sheets. Compos. Struct..

[10] Ge H., Zhao X., Li H., Liang Y., Jiang Y. (2022). Experimental and Numerical Study on CFRP-Strengthened RC Beams. Constr. Build. Mater..

[11] Salem A., Issa M. (2023). Flexural Strengthening of High-Strength and Ultra-High-Strength Concrete Beams with CFRP Sheets. J. Compos. Constr..

[12] Kueres D., Hegger J., Schneider H. (2019). Experimental and Numerical Study on the Flexural Behavior of CFRP-Strengthened RC Beams. Eng. Struct..

[13] Do-Dai N., Trinh T., Pham M. (2022). Numerical Modeling of CFRP-Strengthened Reinforced Concrete Beams. J. Build. Eng..

[14] Thamrin R., Setiadi S., Abdullah M. (2021). Finite Element Analysis of CFRP Strengthening on Reinforced Concrete Beams. Case Stud. Constr. Mater..

[15] Liu Z., Zhao Y., Gu C., Xie Y. (2019). Flexural Strengthening of RC Beams with CFRP Sheets Using Different Bonding Techniques. Constr. Build. Mater..

[16] Al-Rousan R. (2021). The Effect of Elevated Temperatures on the Behavior of RC Beams Strengthened with CFRP Strips. Compos. Struct..

[17] Cao Y., Zhao G., Zhi X. (2021). Flexural Behavior of CFRP-Encased Reinforced Concrete Beams with Different Shear Reinforcements. Constr. Build. Mater..

[18] Qureshi M., Mehmood T., Javed M., Musarat M. (2022). Effect of CFRP Reinforcement Ratio on the Flexural Capacity and Failure Mode of Concrete Beams. Polymers.

[19] Jin Y., Yin S., Wu Z. (2020). Meso-Scale Simulation of CFRP-Strengthened Concrete Beams under Flexural Loading. Polymers.

[20] Amaireh A., Hammoudi A., Abdelrahman A. (2020). Shear Reinforcement Effects on the Behavior of CFRP-Strengthened RC Beams. Case Stud. Constr. Mater..

[21] Hosen M., Rahman M., Islam M. (2019). Flexural Performance of Concrete Beams Strengthened with CFRP Sheets. Constr. Build. Mater..

[22] Iskander M., Mohammadi Y., Javad M. (2020). Experimental and Numerical Investigation on the Placement Effects of CFRP Strip Bonding. Polymers.

[23] Hassan T., Masmoudi R., Neale K. (2018). Shear Rehabilitation of RC Beams with CFRP. Polymers.

[24] COPRAS Method (2022). Comparative Analysis of CFRP Composites Using the COPRAS Method. Polymers.

[25] Abbasi M., Aalami R., Abbasi S. (2022). FE Modeling and Simulation of CFRP Strengthened RC Beams. Polymers.

[26] Sevillano E., Garcia J., Castaneda A. (2016). Comparison of Sensing Technologies for Debonding Detection in CFRP-Strengthened Beams. Polymers.

